# Impact of noradrenergic inhibition on neuroinflammation and pathophysiology in mouse models of Alzheimer’s disease

**DOI:** 10.21203/rs.3.rs-5328229/v1

**Published:** 2024-10-30

**Authors:** Andrew K. Evans, Heui Hye Park, Claire E. Woods, Rachel K. Lam, Daniel Ryskamp Rijsketic, Christine Xu, Emily Chu, Peter Ciari, Sarah Blumenfeld, Laura M. Vidano, Nay L. Saw, Boris D. Heifets, Mehrdad Shamloo

**Affiliations:** Stanford University; Stanford University; Stanford University; Stanford University; Stanford University; Stanford University; Stanford University; Stanford Universityf; Stanford University; Stanford University; Stanford University; Stanford University; Stanford University

**Keywords:** Alzheimer’s Disease, norepinephrine, locus coeruleus, beta-adrenergic receptor, beta-blocker, neuroinflammation, iDISCO+, amyloid beta, microglia, astrocytes

## Abstract

Norepinephrine (NE) modulates cognitive function, arousal, attention, and responses to novelty and stress, and also regulates neuroinflammation. We previously demonstrated behavioral and immunomodulatory effects of beta-adrenergic pharmacology in mouse models of Alzheimer’s disease (AD). The current studies were designed to block noradrenergic signaling in 5XFAD mice through **1)** chemogenetic inhibition of the locus coeruleus (LC), **2)**pharmacologic blocking of β-adrenergic receptors, and **3)** conditional deletion of β1- or β2-adrenergic receptors (adrb1 or adrb2) in microglia.

First, brain-wide AD pathology was mapped in 3D by imaging immunolabeled, cleared 5XFAD brains to assess the overlap between Aβ pathology, reactive microglia, and the loss of tyrosine hydroxylase (TH) expression in the catecholaminergic system. To examine the effects of inhibiting the LC NE system in the 5XFAD model, inhibitory (Gi) DREADD receptors were expressed specifically in LC NE neurons. LC NE neurons were chronically inhibited through the subcutaneous pump administration of the DREADD agonist clozapine-N-oxide (CNO). Plasma and brains were collected for assessment of neuroinflammation and pathology. A separate cohort of 5XFAD mice was chronically dosed with the beta-adrenergic antagonist propranolol or vehicle and evaluated for behavior, as well as post-mortem neuroinflammation and pathology. Finally, we used 5XFAD mice with conditional deletion of either adrb1 or adrb2 in microglia to assess neuroinflammation and pathology mediated by β-adrenergic signaling.

Using iDISCO, light sheet fluorescence microscopy, and novel analyses, we detected widespread microgliosis and amyloid pathology, along with modest TH downregulation in fibers across multiple brain regions, in contrast to the spatially limited TH downregulation observed in neurons. Both chemogenetic inhibition of LC adrenergic signaling and pharmacological inhibition of beta-adrenergic receptors potentiated neuroinflammation without altering amyloid beta pathology. Conditional deletion of adrb1 in microglia did not affect neuroinflammation. Conditional deletion of adrb2 in microglia attenuated inflammation and pathology in females but had no effect in males. Overall, these data support previous observations demonstrating the immunomodulatory effects of beta-adrenergic signaling in the pathophysiology of brain disorders and suggest that adrenergic receptors on cell types other than microglia, such as astrocytes, may predominantly mediate the disease-modifying effects of β-adrenergic agonists in the brain.

## Introduction

Norepinephrine (NE) regulates many brain functions, including learning and memory, responses to novelty, arousal, attention, and energy metabolism (1–8). NE is also a significant regulator of neuroinflammation (9–12). Loss of NE tone with degeneration of the locus coeruleus (LC), the primary source of NE in the brain, is observed with normal aging (13) and exacerbated in neurodegenerative disorders such as Alzheimer’s disease (AD) (3, 12, 14–16). LC neurons exhibit the earliest signs of pathology and degeneration in AD (15, 17–20). LC degeneration, accompanied by a reduction in NE tone, precedes and is thought to contribute to more widespread neurodegeneration in AD (21–24). Involvement of the NE system in the pathophysiology of neurodegenerative disorders has been demonstrated in human clinical studies (25, 26), as discussed in recent reviews (4, 16). For example, a clinical analysis of 4 million Norwegians revealed a reduced risk of neurodegenerative disease in individuals using the β-ADR agonist, salbutamol. Use of the beta-blocker, propranolol, correlated with increased risk and worsening of clinical outcomes in neurodegenerative disease (25). These findings were supported in follow-up studies (26, 27), although another study found no reduced risk of Parkinson’s disease with the use of β2-adrenergic agonists (28). Of note, the potential adverse effects of beta-blocker use on cognitive performance may be masked by pro-cognitive effects related to the antihypertensive and anti-anxiety effects of beta-blockers (29, 30). These findings with CNS active beta-adrenergic agonists and antagonists need further well-controlled clinical investigations to determine their impact on cognitive function and neuroimmune modulation.

Modulation of neuroinflammation and cognitive function by NE is mediated through multiple adrenergic receptor subtypes, including beta-adrenergic receptors (β-ADRs) located on neurons, microglia, and astrocytes (31, 32). Studies, including from our lab, have demonstrated behavioral, immunomodulatory, and disease-modifying effects of β-adrenergic pharmacology in mouse models of AD (10, 11, 33–40). Under experimental conditions, loss of NE tone exacerbates AD-related behavioral deficits, neuroinflammation, and pathology (9, 40, 41). Lesioning of the LC and pharmacological blockade of beta-adrenergic signaling have been shown to potentiate neuroinflammation and worsen behavioral deficits in mouse models of AD (9, 11, 34, 38, 41). We have shown that β-ADR blockers impair learning and memory (11, 33) and amplify neuroinflammation in transgenic mouse models expressing human mutant amyloid precursor protein (APP)(11). Conversely, β-ADR agonists and NE restoration improve behavior and attenuate amyloid beta (Aβ) pathology and neuroinflammation in transgenic APP mice (9, 34, 36). However, the specific cell types and pathways through which NE affects inflammation and pathology in AD remain poorly understood. Identifying these mechanisms is crucial for developing targeted treatments that harness NE’s neuroprotective and anti-inflammatory properties.

Mouse models have been used in mechanistic studies to understand pathology related to AD. However, these models may not recapitulate essential features of the disease, such as neurodegeneration and fiber loss. In recent years, advances in neuroimaging and computational analysis have enabled unprecedented insights into the pathological progression of neurodegenerative diseases. In this study, we employed state-of-the-art techniques to comprehensively map AD pathology across intact 5XFAD mouse brains. The 5XFAD model is well-established for studying Aβ pathology and associated neuroinflammation (42). Brains from 5XFAD and non-carrier (NC) control mice were immunolabeled, optically cleared using iDISCO+, and imaged in 3D via light sheet fluorescence microscopy (LSFM). Here, we quantified and described extensive patterns of Aβ accumulation and corresponding microglial reactivity in the whole brain. Additionally, we characterized TH downregulation in fibers and neurons across 5XFAD brains, substantiating this model for studying the effects of noradrenergic deficiencies on neuroinflammation and pathology.

While genetic deletion studies provide a means to confirm the importance of β-ADR signaling in AD pathology, they are limited by the diverse functions of NE across various brain cell types. To further elucidate the complexity of NE’s modulatory effects on neuroinflammation, we employed a multifaceted approach using the 5XFAD mouse model of amyloidosis. The current study aimed to dissect the contributions of LC-derived NE and β-adrenergic receptor signaling to neuroinflammatory processes in AD. We utilized chemogenetic inhibition of LC neurons, conditional deletion of β1- and β2-ADR (adrb1 and adrb2) specifically in microglia, and pharmacological inhibition of β-adrenergic receptors to assess their impacts on neuroinflammation and Aβ pathology. We demonstrate that chemogenetic inhibition of the LC and chronic administration of the β-adrenergic antagonist propranolol potentiate neuroinflammation in 5XFAD mice, without altering Aβ pathology. In contrast, conditional deletion of adrb1 in microglia had no effect on neuroinflammation, whereas deletion of adrb2 attenuated inflammation, but only in female mice. These findings support the role of adrenergic modulation in AD-related neuroinflammation and point towards the potential involvement of adrenergic receptors on other cell types, such as astrocytic beta-adrenergic receptors, in modulating neuroinflammation in 5XFAD mice.

## Methods

All animal maintenance and experimental procedures were approved by the Stanford University Administrative Panel for Laboratory Animal Care and conformed to the U.S. National Institutes of Health Guide for the Care and Use of Laboratory Animals. Efforts were made to minimize the number of mice used and their suffering. For all studies, mice were group-housed under a reverse light-dark cycle with lights off at 8:30 AM and on at 8:30 PM. Exceptions to group housing were made when individual mice were separated due to in-cage fighting. Food and water were freely available.

### Chemogenetic LC inhibition (DREADD) studies:

**Figure 1:**
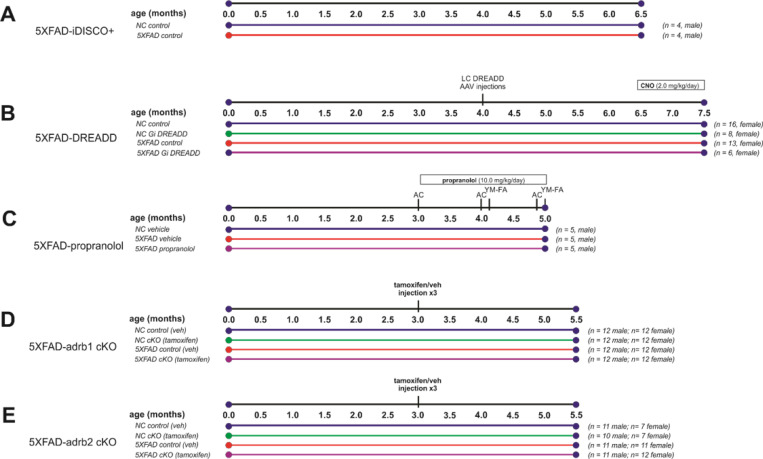
Experimental Designs. **A)** 5XFAD and non-carrier NC controls were aged to 6.5 months old for iDISCO+ and lightsheet imaging of brain pathology. **B-E)** Inhibition of noradrenergic signaling was examined in parallel studies. **B)** Immunological effects of the 5XFAD genotype and chemogenetic inhibition of locus coeruleus (LC) noradrenergic neurons with Designer Receptors Exclusively Activated by Designer Drugs (DREADD) were examined in 5XFAD mice. DREADD agonist clozapine N-oxide (CNO) was administered for 28 days via a pump to activate the inhibitory (Gi) DREADD receptors expressed on LC noradrenergic neurons. Tissue was collected at 7.5 months of age. **C)** Effects of beta-adrenergic receptor blockade with propranolol were examined in 5XFAD mice, and vehicle-treated 5XFAD mice and non-carrier (NC) controls were also compared. Propranolol was administered daily for 2 months (10 mg/kg/day; intraperitoneal). Behavior was assessed in activity chamber (AC) and Y-Maze-forced alternation (YM-FA) assays. Tissue was collected at 5 months of age. **D-E)** Effects of conditional deletion of adrb1 or adrb2 in microglia were studied in male and female 5XFAD and NC control mice. Tamoxifen or the vehicle (veh) control was administered for 3 days to initiate the deletion of ADRB1 or ADRB2 on myeloid lineage cells, including microglia. Gene expression recovers in peripheral myeloid lineage cells with cell turnover and remains absent from microglia in the brain long-term. Tissue was collected at 5.5 months of age.

Mice expressing Cre recombinase from the dopamine beta hydroxylase locus (DBH-cre; derived from B6.FVB(Cg)-*Tg*^(dbh-Cre)KH212Gsat^/Mmucd, MMRRC 036778) were crossed with 5XFAD transgenic mice (RRID:MMRRC_034840-JAX; overexpressing 5 gene mutations related to Familial Alzheimer’s Disease (FAD), 3 mutations in human APP (Swedish, K670N, M671L; Florida, I716V; and London, V717I) and 2 mutations in human presenilin 1, M146L and L286V). This cross resulted in a 5XFAD^+/−^/DBH-Cre^+/−^ mouse line used for pathological endpoints. A cohort of female mice was used in these studies, and group sample sizes and the experimental design are indicated in Figure 1. To induce expression of inhibitory Designer Receptors Exclusively Activated by Designer Drugs (DREADD) receptors on LC neurons, 5XFAD/DBH-Cre mice received bilateral LC injections of AAV expressing Cre-dependent inhibitory (rAAV5-eF1a-DIO-hM4Di-mCherry) or control (rAAV5-eF1a-DIO-mCherry) DREADD viruses (obtained from the Stanford Gene and Virus Vector Core). Bilateral injections were administered at 2 loci targeting the LC (A/P −5.45 mm, M/L +/− 1.3 mm, D/V −3.8 mm & −3.4 mm) with 0.5 μL total per side. Following viral transfection, DBH promoter-dependent Cre recombinase expression resulted in transcription of the DREADD receptor or the control mCherry fluorescent protein tag specifically in NE neurons of the LC. To activate the DREADD receptor, mice were dosed with the designer drug clozapine-N-oxide (CNO) at 2 mg/kg/day via subcutaneous pump administration for 1 month (for experimental timelines, see Figure 1). Pumps (model 1004, Alzet, Cupertino, CA) loaded with CNO, were inserted subcutaneously in the back of each mouse under anesthesia.

### Propranolol studies:

Initial body weight was used as a pseudo-randomization parameter for assigning mice to drug treatment groups (see Figure 1 for Experimental Design). Three-month-old male 5XFAD mice (Jackson, see above) were administered the beta-adrenergic antagonist propranolol (10 mg/kg, i.p.; n=5) or vehicle (i.p.; n=5) daily for 2 months. A NC control group was dosed daily with vehicle (i.p.; n=5). An NC propranolol group was not included in this study as the aim was explicitly to determine the effect of propranolol on AD-related behavior and pathology in 5XFAD mice. Behavior was assessed in the Activity Chamber (AC) and Y-Maze: Forced Alternation (YM-FA).

### Conditional knockout of ADRB receptors in microgliastudies:

For conditional gene deletion studies, mice with a floxed *Adrb1* sequence (gift of Steven Thomas, University of Pennsylvania; C57BL/6J background) or a floxed *Adrb2* sequence (gift of Gerard Karsenty, Columbia; C57BL/6J background) were crossed with mice in which an estrogen receptor ligand-binding-dependent expression of Cre recombinase (CreER) was linked to the expression of the C-X3-C motif chemokine receptor 1 (*Cx3cr1*) gene (Jackson 021160; B6.129P2(Cg)-*Cx3cr1*^*tm2.1(cre/ERT2)Litt*^/WganJ). These crosses resulted in transgenic mouse models (*Adrb1*-flox^+/+^/*Cx3cr1-CreER*^+/−^ or *Adrb2*-flox^+/+^/*Cx3cr1-CreER*^+/−^) with tamoxifen-inducible genetic deletion of *Adrb1* or *Adrb2* specifically in myeloid lineage cells (e.g., microglia and macrophages). Gene deletion specific to microglia was confirmed with RNAScope colocalization of iba1 and adrb1 or adrb2 in control mice but not in mice dosed with tamoxifen. These lines were then crossed with 5XFAD hemizygous mice (Jackson, see above) to examine the effect of deletion of *Adrb1* or *Adrb2* specifically in myeloid lineage cells in 5XFAD mice from both sexes (for Experimental Design and N’s, see Figure 1). To initiate gene deletion, mice were dosed with tamoxifen (Cayman Chemical 13258; 400 mg/kg; p.o.) or corn oil vehicle (Santa Cruz Biotechnology; sc-214761) using a 3-dose regimen, once per week for 3 weeks, designed to maximize Cre-recombinase expression and genetic deletion across all myeloid lineage cells, including microglia.

### Behavioral testing

Behavior was tested in the propranolol study (Figure 1). Mice were handled to obtain body weight and before the beginning of behavioral testing to habituate mice. On the days of behavioral testing, mice were moved to a holding area adjacent to testing rooms prior to the start of assessments. Dosing and tissue collection rooms were separate from housing and behavioral testing rooms. The dosing rooms and the holding area were lit by a red light. Food and water were freely available. All behavioral tests were performed and scored by an experimenter blind to experimental groups.

### Activity chamber (AC)

The AC assessed general locomotor activity and exploration as described previously (43, 44). Mice were placed in one corner of a square Activity Arena (43×43×30 cm; Med Associates Inc., St. Albans, Vermont; Model ENV-515) located inside a dark sound-attenuated chamber (74×60×60 cm) and allowed to explore the arena freely. The movement was tracked by an automated tracking system with three planes of infrared detectors during a 10-minute trial. Parameters measured included distance moved, vertical counts (rearing), and vertical time in the periphery and center of the arena. The periphery was defined as the zone within 5 cm of the arena wall. The arena’s surface was cleaned with 1% Virkon disinfectant between each trial. AC was conducted pre- and post-dosing (CNO or propranolol) at 4- and 8-weeks post-dosing (3, 4, and 5 months of age; propranolol study). Daily dosing occurred at the end of the day after behavioral testing was completed.

### Y-maze: forced alternation (YM-FA)

The YM-FA was used to assess spatial memory. This test is based on the tendency of rodents to preferentially explore a novel environment over a familiar one. In this case, a rodent should prefer to explore a different arm of the maze than the arm they had previously explored. The maze was plastic with 3 arms in a “Y” shape (each arm 40×8×15cm). The test consisted of two 8-minute trials separated by a 1-hour intertrial interval (ITI). To start each trial, mice were placed at the end of one of the arms (Start Arm). During the first trial (Training), mice were only allowed to explore two of the three arms (Familiar Arms). A plastic insert blocked off the third arm (Novel Arm). The Novel Arm was pseudorandomized to avoid any location bias. During the second trial (Testing), this insert was removed, and the mice were allowed to explore all three arms. The trials were recorded with an overhead camera and tracked with Ethovision XT (Noldus Information Technology, Wageningen, Netherlands). Between each trial, the surface of the maze was cleaned with 1% Virkon disinfectant. Y-maze was conducted at 4- and 8-weeks post-dosing (at 4 and 5 months of age).

### Tissue collection

For the propranolol study, mice were dosed with a final dose of propranolol 1 hour prior to tissue collection. For terminal collection for the DREADD, propranolol and conditional knockout studies, mice were deeply anesthetized with isoflurane. Prior to perfusion, whole blood was collected from the right ventricle via cardiac puncture (23 g needle) into lithium heparin-containing vials (BD microtainer plasma tubes, Becton Dickinson 365958) or K3EDTA-containing vials (propranolol study; Greiner Bio-One, MiniCollect Tube Reference #450475) for plasma collection. Plasma tubes were stored on ice before centrifugation within 60 minutes of collection. For perfusion, the right atrium was opened, and mice were transcardially perfused with ice-cold phosphate-buffered saline (PBS; 11.47 g sodium phosphate dibasic and 2.30 g sodium phosphate monobasic per 1 L deionized water; pH 7.4) through a 25 g needle. The perfused brain was removed. The brain was bisected coronally at the level of the mammillary bodies into the forebrain and hindbrain. The forebrain was hemisected. The left forebrain was immediately flash-frozen on dry ice and stored at −80°C for later analysis. The right forebrain and hindbrain were post-fixed with 4% paraformaldehyde in a 15ml conical centrifuge tube (48 hours, 4°C). Afterwards, the right hemispheres were rinsed 2 × 12 hours in phosphate buffer (PB) and cryoprotected for at least 72 hours (until sunk) in 30% sucrose in PB. Fixed brains were then rapidly frozen in isopentane on dry ice. All frozen tissue was stored at −80°C. Whole blood was centrifuged (11K rpm for 2 minutes) for plasma separation, and plasma was aliquoted and frozen on dry ice. Brain and plasma samples were stored at −80°C.

### Aβ ELISA

To determine the amount of soluble and insoluble Aβ(1–40) (Aβ40) and Aβ(1–42) (Aβ42) present in brain tissue, cortical samples were processed as previously described (11, 34). In brief, tissue dissections were weighed and homogenized in 10 volumes of tris-buffered saline (TBS) containing a protease inhibitor cocktail. The samples were centrifuged at 175,000 g for 30 min at 4 °C. The supernatant (TBS-soluble homogenate) was collected and kept at −20 °C. The pellets were re-homogenized in the same volume of TBS-T (TBS/1% Triton X-100 with protease inhibitor cocktail) at 4 °C, centrifuged at 175,000 g for 30 min at 4 °C, and the resultant supernatant (TBS T-soluble homogenate), containing membrane-bound Aβ, was collected and kept at −20 °C. Subsequently, the pellets were homogenized in ice-cold 5M guanidine-HCl in 50 mM Tris (pH 8.0). The homogenates were mixed for 4 hours at room temperature and used to measure insoluble Aβ40 and Aβ42. Finally, amounts of TBS-soluble and guanidine-soluble forms of Aβ were quantified using ELISA kits specific for human Aβ40 and Aβ42 (Invitrogen; #KHB3481 for Aβ40; #KHB3441 for Aβ42) following the manufacturer’s instruction. The final values were normalized to the amount of loaded wet tissue.

### Multiplex mouse cytokine assay

Tissue cytokines were analyzed in brain homogenate from hippocampal (DREADD studies) or frontal cortex (conditional KO studies) dissections using a Luminex 48-plex mouse cytokine assay (ThermoFisher, EPX480–20834-901). The panel includes **Pro-inflammatory cytokines:** BAFF, G-CSF (CSF-3), GM-CSF, IFN alpha (IFNA), IFN gamma (IFNG), IL-1 alpha (IL1A), IL-1 beta (IL1B), IL-2, IL-6, IL-12p70, IL-17A (CTLA-8), IL-18, IL-23, IL-27, IL-31, TNF alpha (TNFA); **Anti-inflammatory cytokines:** IL-4, IL-10, IL-13, IL-19; **Cytokines involved in immune regulation and growth factors:** IL-3, IL-5, IL-7, IL-9, IL-15/IL-15R,IL-22, IL-25 (IL-17E), IL-28, IL-33, LIF, M-CSF, RANKL; **Chemokines:** ENA-78 (CXCL5), Eotaxin (CCL11), GROalpha (CXCL1), IP-10 (CXCL10), MCP-1 (CCL2), MCP-3 (CCL7), MIP-1 alpha (CCL3), MIP-1 beta (CCL4),MIP-2, RANTES (CCL5); **Growth factors/regulators:** Betacellulin (BTC), Leptin, VEGF-A; and **Soluble receptors:** IL-2R, IL-7R alpha (IL17RA), IL-33R (ST2). Briefly, tissue was homogenized in homogenization buffer containing proteinase inhibitor (1:100) by pulling tissue through a 23 g needle (15x) and then sonicated with 3×3 sec pulses. Homogenate was spun at 14,000 g for 10 min, and protein concentrations were determined using the Pierce BCA assay. Samples were diluted to a standard concentration of 6 μg/μL. Brain homogenates were run in singlets on a 96-well plate alongside astandard curve and quality control calibration samples.

### Proteomics

In the propranolol study, proteins from mouse hippocampal dissections were analyzed similar to prior methods (33). Proteomics analyses were performed at the Vincent Coates Foundation Mass Spectrometry Laboratory, Stanford University Mass Spectrometry (SUMS - RRID: SCR_017801). Lysis buffer (5% SDS, 50 mM TEAB, and 1X Protease and Phosphatase Inhibitors) was added to tissue samples, which were then homogenized using a bead mill. The lysate was cleared and transferred for filter-supported digestion. Proteins were reduced with 10 mM DTT at 550°C for 30 min, followed by alkylation with 30 mM acrylamide for 30 minutes at room temperature. Trypsin/LysC protease (0.5 μg; Promega) was added to each sample for overnight digestion at 37°C. After digestion, the reaction was quenched with 1% formic acid, and peptides were eluted and dried. Pierce Quantitative Fluorometric Peptide Assay kit (Thermo Fisher Scientific) was used for peptide quantification. The peptide mixture was dried using a speed vacuum centrifuge before dissolution in reconstitution buffer (2% acetonitrile with 0.1% formic acid). A total of 1 μg of peptides was used for subsequent LC-MS/MS analysis. Mass spectrometry was performed using an Orbitrap Eclipse Tribrid mass spectrometer RRID:022212 (Thermo Scientific, San Jose, CA, USA) with liquid chromatography using an Acquity M-Class UPLC (Waters Corporation, Milford, MA, USA). A flow rate of 300 nL/minute was used, where the mobile phase A was 0.2% formic acid in water and the mobile phase B was 0.2% formic acid in acetonitrile. Analytical columns were prepared in-house with an inner diameter of 100 μm and pulled into to a nanospray emitter using a P2000 laser puller (Sutter Instrument, Novato, CA, USA). The column was packed using C18 Reprosil Pur stationary phase (1.8 μm particle size) to a length of ~25 cm. Peptides were directly injected onto the analytical column and separated using a gradient of 2%–45% solvent B over 80 min, followed by a high-B wash. The mass spectrometer was operated in a data-dependent fashion using CID fragmentation for MS/MS spectra generation. For data analysis, the RAW data files were processed using Byonic v4.1.5 (Protein Metrics, Cupertino, CA, USA) to identify peptides and infer proteins. Proteolysis with Trypsin/LysC was assumed to be semi-specific, allowing for N-ragged cleavage with up to 2 missed cleavage sites. Precursor mass accuracies were held within 12 ppm and 0.4 Da for MS/MS fragments. Cysteine modified with propionamide was set as fixed modifications in the search. Proteins were held to a false discovery rate of 1%, using a standard reverse-decoy technique (45). Pathway analysis for proteomics was conducted using the KEGG mouse 2019 dataset in Enrichr. All proteins showing 2-fold up- or down-regulation (t-test, p < .05) in response to the 5XFAD genotype or propranolol treatment were included in pathway analyses.

### Immunohistochemistry for 6E10, iba1 and TH in brain slices

Immunohistochemistry was used to label and quantify ionized calcium-binding adapter molecule 1 (Iba1), a marker for microglia/macrophages, Aβ (6E10), and tyrosine hydroxylase (TH), alongside a nucleic acid stain, 4’,6-diamidino-2-phenylindole dihydrochloride (DAPI). Fixed brains were serially sectioned (at −18°C using a Microm HM-550 cryostat) in a coronal plane, creating six series of sections spanning the rostrocaudal axis. For the forebrain, sections were 40 μm thick with 240 μm intervals between sections within each series, and for the hindbrain, sections were 30 μm thick with 180 μm intervals. Sections were stored in cryoprotectant buffer (30% ethylene glycol, 20% glycerol in 0.05M phosphate buffer, pH 7.4). Multilabel fluorescent immunohistochemistry was performed on one series of brain sections to double-label Iba1 and 6E10 in the rostral hippocampus (from 0.26 to −2.92 mm Bregma) (46) and on another series for TH labeling through the LC (from −5.34 to −5.80 mm Bregma) (46). Free-floating sections were incubated at room temperature in 24-well tissue culture plates gently shaken on an orbital shaker. All rinses were 15 min unless stated otherwise. Sections were rinsed three times in 0.05 M PBS, and then preincubated for 1 h in PBS containing 1% Triton X-100 (PBST) and 3% bovine serum albumin. Sections were incubated for 18 h with a goat anti-iba1 primary antibody (Abcam, ab5076, 1:1000), a mouse anti-6E10 primary antibody (Biolegend, 803001, 1:1000; binds to amino acid residues 1–16 of Aβ), or a chicken anti-TH primary antibody (Abcam, ab76442, 1:2000) in 0.3% PBST and 1% bovine serum albumin. Following 3 PBS rinses, sections were incubated for 2 hours in IgG and/or IgY secondary antibodies, each diluted 1:250 in PBS (Cy5-conjugated AffiniPure donkey anti-chicken, 703–175-155; Cy3-conjugated AffiniPure donkey anti-goat, 705–165-147; 488-conjugated AffiniPure donkey anti-mouse, 715–545-151; Jackson Immunoresearch, Bar Harbor, ME). The secondary incubation included DAPI (D9542, Sigma-Aldrich, St. Louis, MO) diluted 1:5000. Free-floating sections were then rinsed 3 times in PB, mounted on clean glass slides, and allowed to air-dry to affix sections to slides immediately prior to coverslipping with polyvinyl alcohol mounting medium with DABCO antifade (10981, Sigma-Aldrich).

### LC NE stereological cell counts

LC NE cells were counted using stereology methods by an experimenter blind to treatment groups. Three sections, 180 μm apart, through the rostral to mid-rostrocaudal LC, were selected for counting. TH-positive neurons were counted via the optical fractionation method using a Zeiss AxioImager M1 microscope (Carl Zeiss) and Stereo Investigator software (version 2019.1.1, MBF Bioscience, Vermont). The sampling grid was set to cover 20% of the LC.

### Whole-brain immunostaining, clearing, imaging and quantification

#### Immunofluorescence staining and iDISCO+

A separate cohort of male 5XFAD mice and NC control mice (Jackson Labs, RRID:MMRRC_034840-JAX) were used for brain-wide imaging studies. Mice were aged to 6.5 months before being anesthetized with 4% isoflurane and intracardially perfused with PBS followed by 4% PFA. Brains were removed, hemisected, and post-fixed overnight. Immunofluorescence staining and iDISCO+ were performed as previously described (47, 48). The hemispheres were dehydrated in a methanol series, bleached in methanol with 5% hydrogen peroxide, rehydrated, permeabilized, and blocked. They were then incubated with primary antibodies for 7 days. In the left hemispheres, microglia and Aβ plaques were labeled with goat anti-Iba1 (Abcam; ab5076; 1:100) and mouse anti-6E10 (Biolegend; 803001, 1:100) antibodies, respectively. In the right hemispheres, TH was labeled with rabbit anti-TH (Sigma Millipore; AB152; 1:200). After washing thoroughly, hemispheres were incubated with secondary antibodies for 7 days (Jackson Immuno Research: donkey anti-goat Cy3, 705–165-147, 1:100; donkey anti-mouse Cy5, 715–175-151, 1:100; donkey anti-rabbit Cy3, 711–165-152, 1:100). Tissue was repeatedly washed before being embedded in 1% low melting point agarose (ThermoFisher; R0801), dissolved in PBS with 0.02% sodium azide. The brains were dehydrated again, incubated in dichloromethane, and cleared/stored in dibenzyl ether.

#### Light sheet microscopy (LSFM)

Cleared brains were imaged in 3D as described (48). Briefly, brains were mounted with a c-clamp and immersed in ethyl cinnamate for imaging with a Zeiss Lightsheet 7. 3D images of entire hemispheres consisted of tiled z-stacks (~800 × 688 pixels each), which were stitched together (10% overlap). Light sheets (10 μm thick) were pivot scanned and averaged together. A 2.5x detection objective captured images with 0.52x zoom (3.52 μm resolution). Z-steps were 5 μm for the left hemisphere and 6 μm for the right hemisphere. For concurrent imaging of autofluorescence and immunolabeling of 6E10 and Iba1, autofluorescence was excited at 488 nm (10% power), and emissions passed through a 505–530 nm filter. Cy3 was excited with 561 nm light (30% power), and emissions passed through a 585 nm long pass filter. Cy5 was excited with a 638 nm laser (5% power), with emissions also passing through a 585 nm long pass filter. For TH staining, the 488 nm laser was set to 6% power, and emissions passed through a 505–545 nm filter. The 561 nm laser (2% power) evoked Cy3 fluorescence, passing through a 575–615 nm filter. The exposure time was 50 ms for all wavelengths.

#### Atlas registration

Processing was automated using UNRAVEL (48). The newer, Python-refactored code is available here (github.com/b-heifets/UNRAVEL/) with documentation here (b-heifets.github.io/UNRAVEL/index.html). In some instances, older scripts were used for this study, (github.com/b-heifets/UNRAVEL/blob/feature), with additional information here (github.com/b-heifets/UNRAVEL/blob/feature/Heifets_lab_guides/UNRAVEL_guide_Heifets_lab_021623.pdf). The autofluorescence channel of stitched z-stacks was downsampled 8x. Midline cuts were digitally corrected using 3D Slicer by trimming excess contralateral tissue and/or adding missing tissue to improve registration quality. Brain tissue was masked using Ilastik project (pixel classification workflow) to zero out external voxels for registration at 50 μm resolution. Registration and warping were performed with modified scripts from MIRACL (49) using an iDISCO+/LSFM-specific average template brain (50) (25 μm resolution; Allen Mouse Brain Common Coordinate Framework v3 with region labels from 2017). Registration quality was visually inspected with ITK-SNAP.

#### Voxel-wise statistics

Immunofluorescence (IF) images were background-subtracted using the rolling ball method (pixel radius 20) to remove autofluorescence and normalize background intensities. TH-IF images were warped to atlas space using transformation matrices from the registration process. Due to the mouse origin of the 6E10 antibody, capillary staining varied with perfusion quality. Ilastik was trained to segment this non-specific staining in the raw IF images, and the resulting mask was applied to remove these artifacts in the full-resolution background-subtracted 6E10-IF images. Both non-reactive and reactive microglia were present in 5XFAD brains, with reactive microglia often aggregating around amyloid-beta plaques. For voxel-wise analyses, Ilastik was trained to segment these aggregates and individual reactive microglia using raw Iba1-IF training images. Ilastik was trained to identify reactive microglia using images from both control and 5X brains (three 2D images per brain). Pixels of presumed reactive microglia and background (i.e., pixels not corresponding to reactive microglia) were sparsely labeled by the experimenter during training. Microglia were considered reactive either when they were aggregated (e.g., surrounding A-beta plaques) and/or iba1-immunoreactivity was higher than in the non-reactive microglia from the training set (e.g., in control brains). Ilastik training focused on minimizing the detection of resting microglia in control brains while maximizing the detection of reactive microglia in 5XFAD brains. Reactive microglia were segmented in raw Iba1-IF images for all brains. Separately, autofluorescence was removed from each Iba1-IF image by rolling ball background subtraction. To preserve signal from reactive microglia in the background-subtracted Iba1-IF images, segmentations from Ilastik for each brain were used to zero out voxels not corresponding to reactive microglia. Masked and background-subtracted 6E10-IF and Iba1-IF images were then warped to atlas space.

Atlas space images individually z-scored using a brain mask that excluded the ventricles, olfactory bulb, and undefined regions. IF images were smoothed using fslmaths (100 μm kernel) from FSL (FMRIB software library). Voxel-wise comparisons were performed with FSL’s randomise_parallel command (18,000 permutations) using a t-test design. Adjustments to p values for multiple comparisons were made with false discovery rate correction, defining clusters of significant voxels. The most stringent q value-producing clusters was used for each immunolabel (q < 0.2 for 6e10 and Iba1 and q < 0.4 for TH). This corresponded to an adjusted p value threshold of 0.025 for 6e10 and Iba1, and 0.011 for TH. Cluster extent thresholding filtered out small clusters likely representing noise, with empirically determined minimum cluster sizes of 400 voxels for 6E10 and Iba1, and 100 voxels for TH. For TH clusters, masks of regions with dopaminergic or noradrenergic neurons were used for TH+ cell density measurements during cluster validation. Otherwise, the full extent of the clusters was used for label density measurements.

#### Cluster validation

Cluster validation was conducted as described (48). To confirm that intensity-based differences in voxel-wise analyses reflect a difference in label or cell density, clusters were warped to tissue space and scaled to full resolution. Ilastik was trained to segment Aβ plaques in raw 6E10-IF images, reactive microglia in Iba1-IF images, and TH+ fibers and cells in TH-IF images. Label densities in clusters were calculated for 6E10+ aggregates, reactive microglia, and TH+ fibers as ((segmented voxel volume) / (cluster volume)) * 100. TH+ cells were counted using the cc3d.connected_components() function with a connectivity of 6, and the cell count was divided by the cluster volume to calculate cell density. Clusters were considered valid if an unpaired, one-tailed t-test showed a significant difference in cell or label densities between groups. Images with valid clusters were visualized in 3D (DSI Studio). Regional composition was determined by multiplying binarized valid cluster images with the atlas and represented in sunburst plots (Flourish). Information on valid clusters is summarized in Supplementary Table S10, with region abbreviations defined in Supplementary Table S9. The top four regions’ volumes were calculated and reported if they constituted more than 80% of the total cluster volume; otherwise, subregions were collapsed into parent regions.

#### General statistics

Unless stated otherwise (see Methods - LSFM), statistical analyses were performed with GraphPad Prism 10.2. Repeated measures, one-way or two-way analyses of variance were followed by post-hoc comparison of select treatment groups with appropriate tests correcting for multiple comparisons. Significance was reported relative to p < 0.05, but results with effects approaching this threshold are also discussed as relevant trends (51, 52).

## Results

### Unbiased whole-brain analysis of the distribution of Aβ aggregates, reactive microglia, and pathology within the catecholaminergic system in 5XFAD and NC control mice

**Figure 2:**
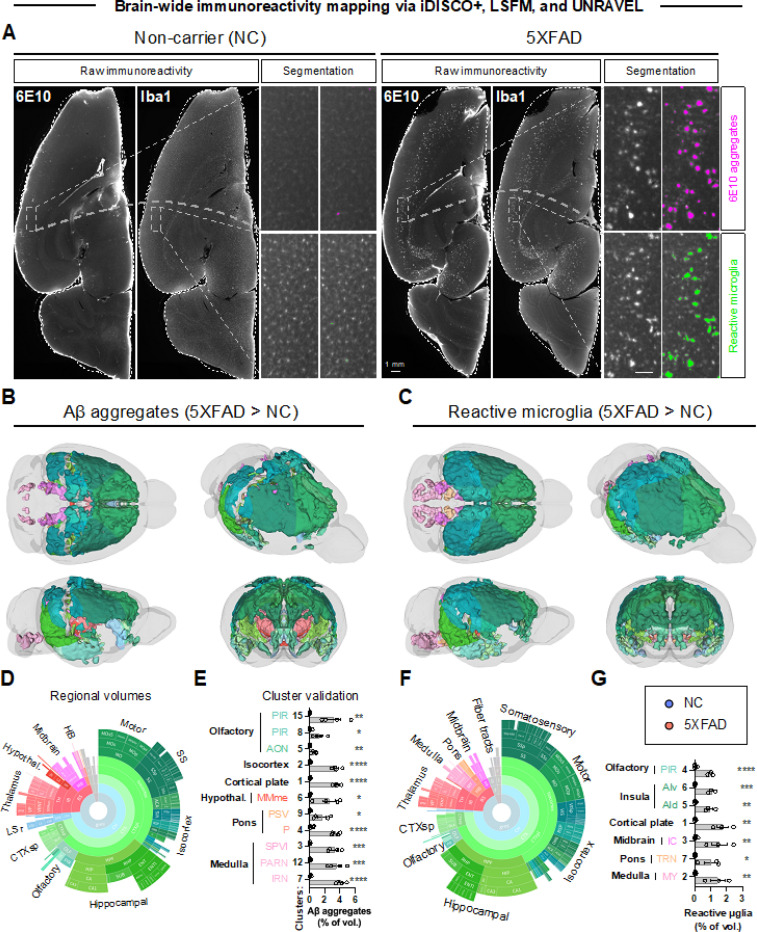
Mapping Aβaggregation and microglial reactivity in 5XFAD mouse brains. Brains from aged 5XFAD mice (6.5 months) and non-carrier (NC) controls were immunostained and cleared with iDISCO+. Aβ plaques (anti-6E10) and microglia (anti-Iba1) were labeled in the left hemispheres. Brains were imaged in 3D with LSFM and analyzed using UNRAVEL. **A)** Examples of raw immunoreactivity are shown with the dashed outlines representing the outline of the registered atlas (white). The scale bar in the zoomed inset is 100 μm. Ilastik segmentations are shown for Aβ plaques and reactive microglia for 5XFAD mice and NC controls. For voxel-wise analyses, artifacts (edges and capillaries) were masked in the background-subtracted 6E10-immunofluorescence images, and voxels not associated with reactive microglia were masked the background-subtracted Iba1-immunofluorescence images. The resulting images were then warped to atlas space, z-scored, and smoothed for intensity-based voxel-wise comparisons, followed by false discovery rate correction (q < 0.2). Clusters of significant voxels were warped back to full-resolution tissue space to measure label densities (the volume of segmented 6E10 aggregates / cluster volume * 100 or the volume of segmented reactive microglia / cluster volume * 100). Clusters were considered valid if unpaired one-tailed t-tests confirmed differences between the groups. **B,C)** Valid clusters are shown in 3D brain models with Allen brain atlas coloring. For display purposes, unilateral cluster maps were mirrored. **D and F)** The regional composition of valid clusters by volume is shown in sunburst plots (outer rings represent subregions, whereas inner rings correspond to parent regions). **E and G)**Bar graphs summarize data for all valid clusters. n=4 for all groups, except for 5XFAD iba1 where n=3 (one sample was excluded due to a technical error in sample preparation). Mean ± SEM. *p < 0.05, **p < 0.01, ***p < 0.001, ****p < 0.001. Supplemental Table S9 defines region abbreviations, and Supplemental Table S10 reports the significance, volumes, positions, and regional composition of valid clusters. Numbers ([‘2/3’,reports the significance, volumes, positions, and regional composition of valid clusters. Numbers ([‘2/3’,’5’, ‘6a’]) = cortical layers. Additional abbreviations not defined in Supplemental Table S9 include: {‘c’:’central nucleus’, ‘d’: ‘dorsal nucleus or dorsal’, ‘e’: ‘external nucleus’, ‘me’: ‘median’, ‘mot’: ‘motor related’,’sen’: ‘sensory related’, ‘v’: ‘ventral’}.

**Figure 3:**
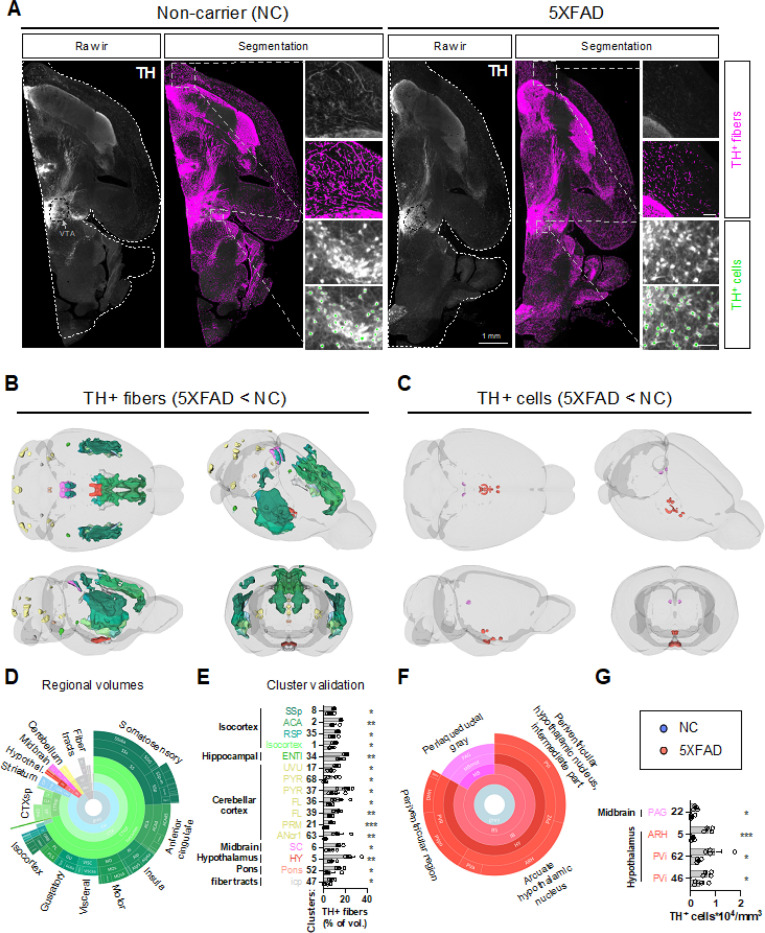
Fewer TH+ fibers and cells are detected in 5XFAD mouse brains. Tyrosine hydroxylase (TH) was immunolabeled in the right hemispheres. Brains were imaged in 3D with LSFM and analyzed using UNRAVEL. **A)** Examples of raw immunoreactivity (ir) are shown for 5XFAD mice and non-carrier (NC) controls, with dashed outlines indicating the registered atlas (white) and the ventral tegmental area (black). Scale bars are 100 μm. Autofluorescence was removed from immunofluorescence images. The resulting images were warped to atlas space, z-scored, and smoothed for intensity-based voxel-wise comparisons, followed by FDR correction (q < 0.4). Clusters of significant voxels were warped back to full-resolution tissue space to measure label densities (the volume of segmented TH+ fibers / cluster volume * 100) or, using the subset of clusters overlapping regions with catecholaminergic neurons, cell densities (TH+ cells / cluster volume). Clusters were considered valid if unpaired one-tailed t-tests confirmed differences in TH+ fiber or cell densities between the groups. **B and C)** Valid clusters are shown in 3D brains with Allen brain atlas coloring. For display purposes, unilateral clusters were mirrored. **D and F)** The regional composition of valid clusters by volume is shown in sunburst plots. **E and G)** Bar graphs summarize data for all valid clusters. n=4 for both groups. *p < 0.05, **p < 0.01, ***p < 0.001. Supplemental Table S10 summarizes the significance, volumes, positions, and regional composition of valid clusters. Numbers ([‘1’, ‘2/3’, ‘3’, ‘4’, ‘5’]) indicate cortical layers, except ANcr1 standsfor Crus 1. Additional abbreviations not defined in Supplemental Table S9 include: {‘agl’: ‘agranular’, ‘l’:’lateral’, ‘p’: ‘primary’, ‘p-bfd’: ‘primary (barrel field)’, ‘p-tr’: ‘primary (trunk)’, ‘po’: ‘preoptic’, ‘s’: ‘supplemental’,’sg’: ‘superficial gray layer’, ‘v’: ‘ventral’, ‘zo’: ‘zonal layer’}.

To characterize the extent of pathology in the 5XFAD model, we analyzed the brain-wide distribution of Aβ plaques, reactive microglia, and catecholaminergic system alterations in a cohort of 6.5-month-old 5XFAD mice and NC controls using immunofluorescence staining, optical clearing, and LSFM. Voxel-wise statistical analyses and false discovery rate corrections identified clusters of significant voxels representing differences in Aβ deposition, reactive microglia, and TH immunoreactivity. These clusters were warped to tissue space to precisely measure the densities of segmented Aβ plaques, reactive microglia, TH+ fibers, and TH+ cells. Data for valid clusters with confirmed differences between 5XFAD mice and NC controls are shown in Figures 2 and 3, Supplemental Figure S1, and Supplemental VideoS1. Supplemental Table S10 summarizes the volume, location, and regional composition of each valid cluster. Supplemental Table S9 defines region abbreviations. Mapping Aβ plaques revealed extensive aggregation across the brain (Figure 2). The largest cluster (cluster 1), accounting for 60.9% of total cluster, spanned several regions including the hippocampal formation (e.g., entorhinal areas, subiculum,CA1, CA3, and dentate gyrus), olfactory areas (e.g., piriform area, post-piriform transition area, and cortical amygdalar areas), isocortex (visual, ectorhinal, temporal, insular, retro splenial, perirhinal, parietal, auditory, and visceral areas), cortical subplate (basolateral/basomedial/posterior amygdala and endopiriform nuclei), thalamus (e.g., sensory-motor nuclei in the ventral group of the dorsal thalamusand several polymodal association nuclei), hypothalamus (e.g., zona incerta), midbrain (e.g., motor-related parts of the superior colliculus, midbrain reticular nucleus, and anterior pretectal nucleus), and fiber tracts (lateral/medial forebrain bundle systems). Cluster 2 (36.6% of total cluster volume) was primarily isocortical (motor, somatosensory, anterior cingulate, retro splenial, orbital, prelimbic, visual, parietal, auditory, infralimbic, frontal pole, and agranular insula). However, a small portion was in theolfactory regions (anterior olfactory nucleus, dorsal taenia tecta, dorsal peduncular area), striatum(lateral septal complex [rostroventral part] and the nucleus accumbens), and pallidum (e.g., medialseptum and substantia innominata). In the isocortical portion of all valid clusters, Aβ aggregates weremost prevalent in layer 5 (46.0%) and layer 6a (39.4%), revealing extensive amyloidosis in deeper cortical layers. This finding aligns with the original characterization of 5XFAD mice, which identified the highe stplaque burden in the subiculum and cortical layer 5, resulting in the loss of pyramidal neurons in these regions by 9 months of age (42).

Aggregates of reactive microglia were commonly observed surrounding Aβ plaques, and the overall distribution of reactive microglia closely matched the pattern of Aβ accumulation (Figure 2). The largest cluster (92.7% of total cluster volume) was 81.0% cortical and overlapped the same list of regions mentioned above. Across cortical regions of valid clusters, 44.2% and 44.6% were localized to layers 5 and 6a, respectively. The second largest cluster (6.5% of total cluster volume) was restricted to the hindbrain. Most of its volume was in motor-related reticular nuclei in the medulla (intermediate, parvicellular, medullary, and gigantocellular). It also was in sensory regions of the medulla (spinal nucleus of the trigeminal), as well as behavioral state, motor, and sensory portions of the pons. Every hindbrain region with Aβ plaques also had reactive microglia. Quantitative analysis of Aβ plaques and reactive microglia densities in these clusters underscored the extensive pathology in the 5XFAD model, with substantial increases in plaque burden and microglial activation compared to controls.

We also examined the loss of TH expression, an indicator of catecholaminergic (NE and dopamine) neuron health. There was a notable reduction in TH+ fibers and cells in specific brain regions (Figure 3). Downregulation of TH in fibers was observed in the isocortex (e.g., somatosensory, anterior cingulate, agranular insula, motor, visceral, gustatory, prelimbic, orbital, and retrosplenial areas), cortical subplate (e.g., dorsal endopiriform nucleus, claustrum, and lateral amygdala), caudoputamen, hypothalamus, midbrain (superior colliculus), cerebellar cortex, and fiber tracts (e.g., corpus callosum). Across cortical regions of valid clusters, TH downregulation in fibers occurred 41.0% in layer 5 and 44.2% in layer 6a, consistent with the predominant presence of plaques and microglial reactivity in these deeper layers. Though less extensive, the distribution of TH downregulation in fibers mirrors Aβ plaques and microglial reactivity, hinting at localized axonal damage from these pathological factors.

In contrast to widespread TH downregulation in fibers, TH downregulation in neuronal cell bodies was spatially confined, affecting hypothalamic nuclei (arcuate nucleus and anterior, intermediate, and preoptic parts of the periventricular nucleus) and the periaqueductal grey. Surprisingly, these regions were not identified in analyses of Aβ plaques or microglial reactivity, suggesting distal damage to these neurons. Together, these findings map Aβ plaque deposition and microglial reactivity, while also suggesting the potential vulnerability of specific catecholaminergic neurons and axonal projections in 5XFAD mice.

### Chemogenetic inhibition of the LC potentiated neuroinflammation in 5XFAD mice but did not impact Aβ pathology

**Figure 4:**
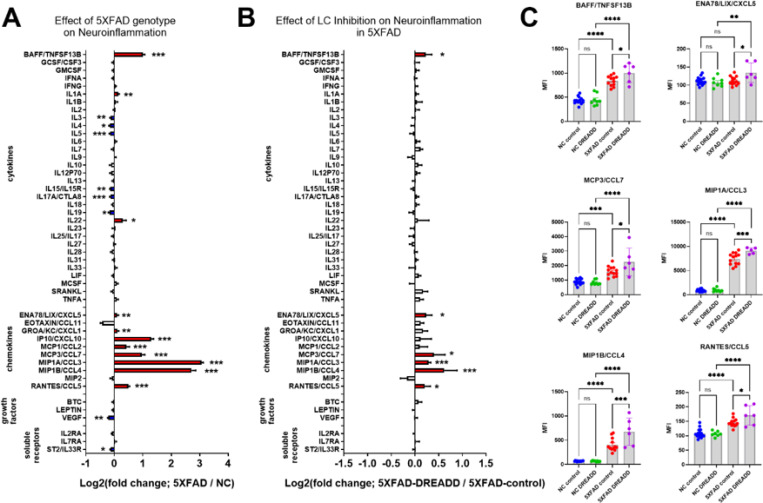
Chemogenetic inhibition of locus coeruleus (LC) noradrenergic neurons with DREADD receptors potentiates CNS inflammation in the 5XFAD mouse model of amyloidosis. Log2-fold change graphs depict the effects of **A)** 5XFAD (5XFAD/non-carrier (NC); n=19) and **B)** LC inhibition (5XFAD-DREADD/5XFAD-control; n=6) in 5XFAD mice on a panel of inflammation-related markers. **C)** Bar graphs display raw data mean fluorescence intensity (MFI) for proteins affected by LC inhibition, as indicated in panel **B**. For A)* indicates main effects. For **B)** and **C)**, * indicates post-hoc Sidak’s comparison of means following two-way ANOVA (5XFAD x DREADD). *p < 0.05, **p < 0.01, ***p < 0.001, ****p < 0.0001.

In the same experiment with mice aged to 7.5 months (Figure 1), we investigated the effects of the 5XFAD genotype and noradrenergic tone loss on inflammation markers. To suppress noradrenergic tone in the 5XFAD brain, LC NE neurons were selectively inhibited using chemogenetics (rAAV5-eF1a-DIO-hM4Di-mCherry in 5XFAD/DBH-Cre mice). After one month of chronic CNO treatment, hippocampal samples were analyzed using a Luminex cytokine panel. Two-way ANOVAs revealed a main effect of 5XFAD on the brain concentrations of proteins including: BAFF, IL1A, IL3, IL4, IL5, IL15/IL15R, IL17A/CTLA8, IL19, IL22, ENA78/LIX/CXCL5, GROA/KC/CXCL1, IP10/CXCL10, MCP1/CCL2, MCP3/CCL7, MIP1A/CCL3, MIP1B/CCL4, RANTES/CCL5, VEGF, and ST2/IL33R (see Figure 4A and Table S1 for complete ANOVA results). Several pro-inflammatory chemokines were significantly upregulated in 5XFAD mice (e.g., IP10/CXCL10, MCP1/CCL2, MCP3/CCL7, MIP1A/CCL3, MIP1B/CCL4, RANTES/CCL5) compared to NC controls, suggesting potential monocyte recruitment and microglial proliferation. Additionally, pro-inflammatory cytokines such as BAFF and IL1A were upregulated, while anti-inflammatory cytokines (IL4 and IL19) were downregulated, further indicating an inflammatory state. Chemogenetic inhibition of the LC in female 5XFAD mice (Figure 4B, Figure 4C, and Supplementary Table S1) potentiated the expression of several inflammatory markers upregulated in 5XFAD mice, including BAFF, ENA78/LIX/CXCL5, MCP3/CCL7, MIPA1/CCL3, MIP1B/CCL4, and RANTES/CCL5, compared to rAAV5-eF1a-DIO-mCherry 5XFAD/DBH-Cre controls. To determine whether LC inhibition with DREADD impacted Aβ pathology, soluble and insoluble Aβ40 and Aβ42 levels were quantified in frontal cortical tissue using ELISA. Chemogenetic inhibition of the LC did not affect cortical Aβ concentrations in female 5XFAD mice (Supplementary Figure S2).

### Propranolol potentiates neuroinflammation and systemic inflammation in 5XFAD mice but did not affect Aβpathology.

**Figure 5:**
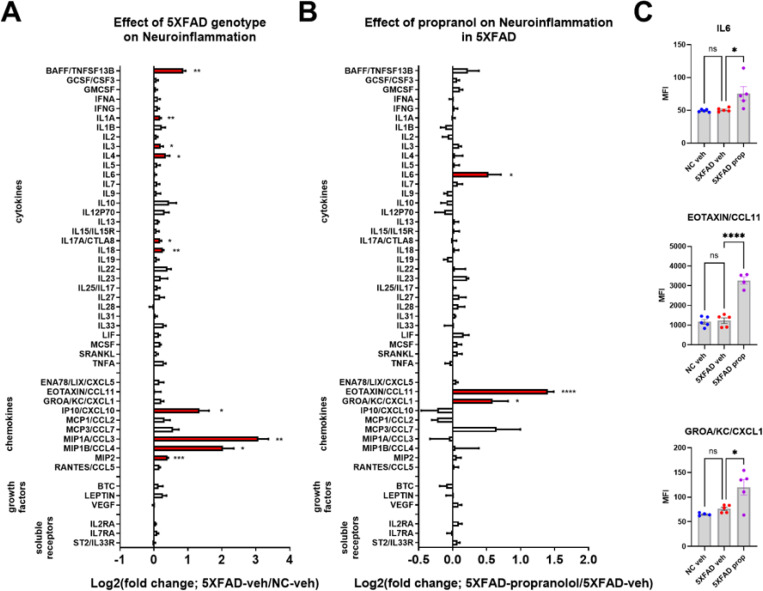
Pharmacological antagonism of beta-adrenergic receptors with propranolol (prop) potentiates CNS inflammation in the 5XFAD mouse model of amyloidosis. Log2-fold change graphs depict the effects of **A)** 5XFAD in vehicle (veh)-treated mice (5XFAD-veh/non-carrier (NC)-veh; n=5) and **B)** propranolol in5XFAD mice (5XFAD-prop/5XFAD-veh; n=5). **C)** Bar graphs show raw data from proteins affected bypropranolol, as indicated in panel **B**. *p < 0.05, **p < 0.01, ***p < 0.001, ****p < 0.0001; Sidak’s post-hoccomparison of means following one-way ANOVA.

To determine the effects of beta-adrenergic blockade on inflammation, pathology, and behavior in 5XFAD mice, the adrb1/adrb2 antagonist propranolol was administered to male 5XFAD mice daily from 3 to 5 months of age. At 5 months, brain dissections (including frontal cortex and striatum) and plasma were assessed for inflammatory markers using a Luminex cytokine panel. As in the DREADD studies, 5XFAD control mice showed robust neuroinflammation with respect to NC controls, with elevated levels of BAFF, IL1A, IL3, IL4, IL17a, IL18, IP10, MIP1A, MIP1B, and MIP2 (Figure 5A and Supplemental Table S2). Consistent with exacerbated inflammation from beta-adrenergic receptor antagonism, propranolol upregulated a few pro-inflammatory markers in 5XFAD mice (IL6, Eotaxin, GROA/KC/CXCL1; Figure 5B and 5C and Supplemental Table S2).

In plasma from the 5XFAD-propranolol study, leptin was the only systemic marker significantly elevated in 5XFAD-vehicle mice compared to NC-vehicle controls, though several other markers approached significance (Supplementary Figure S3A and Supplementary Table S3). Propranolol induced a few markers in the plasma of 5XFAD mice (GCSF, GROA/KC/CXCL1, and MCP3/CCL7) and reversed the leptin increase observed in 5XFAD mice (Supplementary Figure S3B,C and Supplementary Table S3).

Analysis of Aβ pathology in the frontal cortex revealed no impact of propranolol on Aβ pathology (soluble and insoluble Aβ42; Supplemental Figure S4). Additionally, no behavioral effects of propranolol were detected in 5XFAD mice in either the Activity Chamber or forced alternation Y-Maze (Supplementary Figure S5).

**Figure 6:**
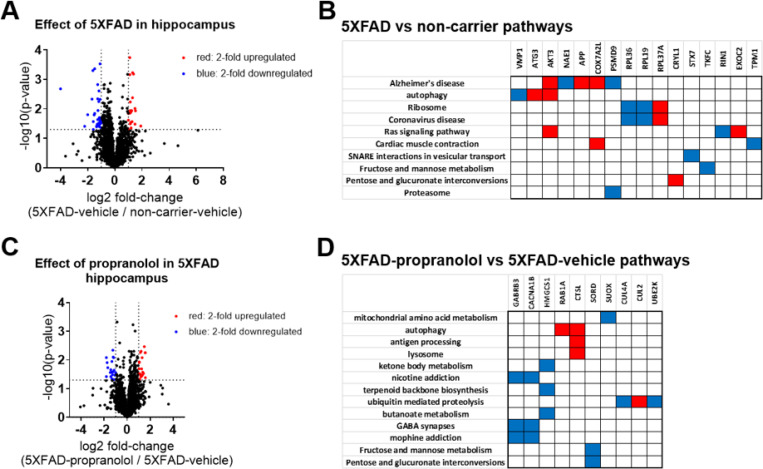
Pharmacological inhibition of beta-adrenergic receptors with propranolol alters the proteome in the 5XFAD mouse model of amyloidosis. Volcano plots depict the effects of 5XFAD and propranolol on the hippocampal proteome, illustrating fold change and significance for detected proteins. Over 3000 proteins were identified in the hippocampus. **A)** 58 proteins were upregulated (red) or downregulated (blue) with the 5XFAD genotype (5XFAD-veh/NC-veh), using a fold change of 2 and a p-value cutoff p < 0.05 (-Log10(p-value) > 1.3). **B)** Clustergram analysis identified proteins modulated by 5XFAD in specific pathways. Blue indicates downregulation and red indicates upregulation. KEGG analysis revealed that pathways impacted by 5XFAD in the hippocampus include autophagy, metabolic pathways, and neurodegeneration-related pathways. **C)** 41 proteins were upregulated (red) or downregulated (blue) by propranolol, using the same thresholds. **D)** Clustergram analysis, based on KEGG, identified propranolol-modulated proteins in specific pathways, including metabolic pathways and those related to autophagy and lysosomes.

Proteomics analyses from the frontal cortices of 5XFAD-propranolol, 5XFAD-vehicle, and NC-vehicle treated mice revealed changes in protein expression due to 5XFAD genotype relative to NC controls as well as effects of propranolol in 5XFAD mice relative to vehicle-treated 5XFAD mice (Figure 6). In 5XFAD mice relative to NC controls, 22 proteins were upregulated and 36 were downregulated (p < 0.05 and at least a 2-fold difference). Pathway analyses of proteins modulated by genotype (5XFAD) revealed pathways related to Alzheimer’s disease, autophagy, and metabolism (Figure 6B). In the frontal cortex, propranolol treatment in 5XFAD mice led to the upregulation of 22 proteins and downregulation of 19 proteins (Figure 6C). Pathway analyses of proteins modulated by propranolol highlighted pathways related to metabolism, autophagy, and GABAergic synaptic transmission (Figure 6D).

### Conditional knockout of adrb2 in microglia attenuatedinflammation in female but not male mice

**Figure 7:**
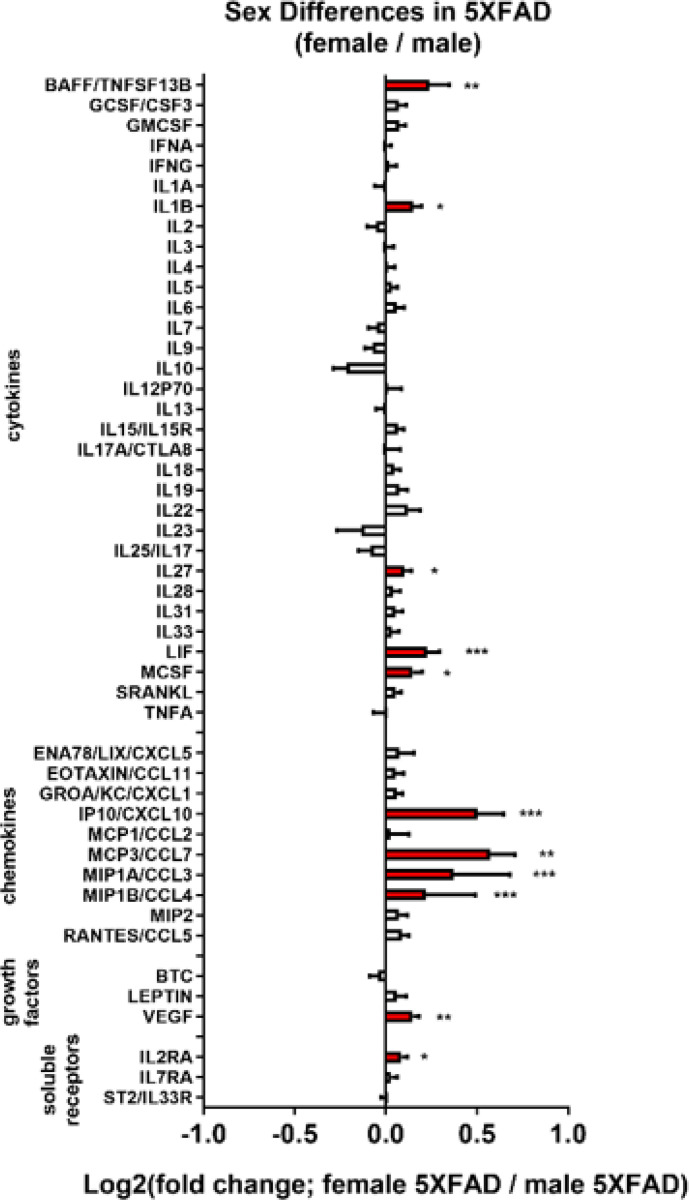
Sex differences in 5XFAD neuroinflammation. The log2-fold change graph depicts the effects of sex in 5XFAD mice (female-5XFAD/male-5XFAD) from the adrb2 cKO study on a panel of inflammation-related markers. *p < 0.05, **p < 0.01, ***p < 0.001, ****p < 0.0001; main effect of sex with two-way ANOVA (Sex x Treatment).

Sex *differences in 5XFAD mice*: Both male and female 5XFAD mice were used to examine the effects of conditional knockout (cKO) of adrb1 or adrb2 in microglia. Clear sex differences in neuroinflammation in 5XFAD mice were observed. Female 5XFAD mice had more extensive neuroinflammation (Figure 7; data from adrb2 study) and greater Aβ40 levels (see Figure 10; data from adrb2 study) than males. Two-way ANOVA revealed main effects of Sex with elevated BAFF, IL1B, IL27, LIF, MCSF, IP10/CXCL10, MCP3/CCL7, MIP1A/CCL3, MIP1B/CCL4, VEGF, and IL2RA in female mice relative to males (Figure 7 Supplementary Table S8 and for ANOVA and post-hoc statistics).

**Figure 8:**
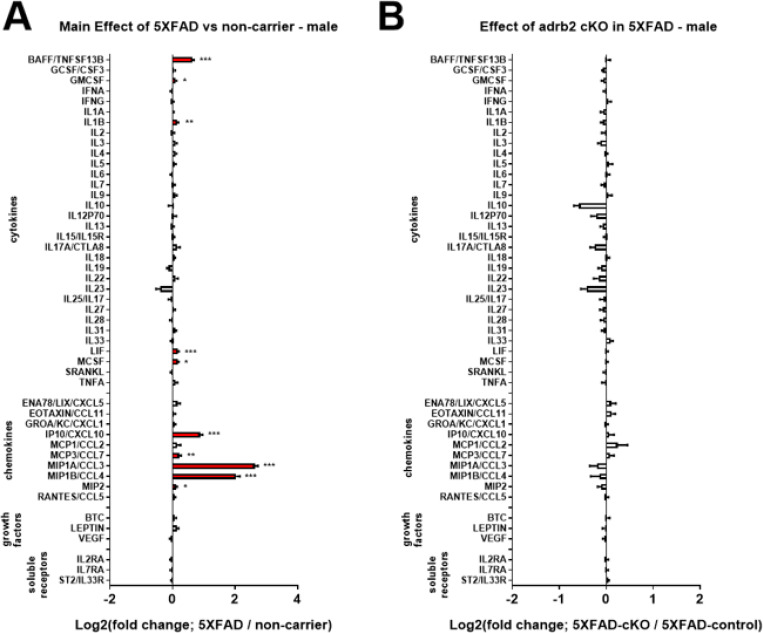
Effect of conditional knockout of adrb2 in microglia in male 5XFAD and non-carrier mice. Log2-fold change graphs depict the effects of **A)**5XFAD (5XFAD/non-carrier; n=20) and **B)**adrb2 cKO in microglia (5XFAD-cKO/5XFAD-control; n=10) in male 5XFAD mice on a panel of inflammation-related markers. For A)* indicates a main effect of 5XFAD. For B)* indicates post-hoc Sidak’s comparison of means following two-way ANOVA (5XFAD x cKO). *p < 0.05, **p < 0.01, ***p < 0.001, ****p < 0.0001.

**Figure 9:**
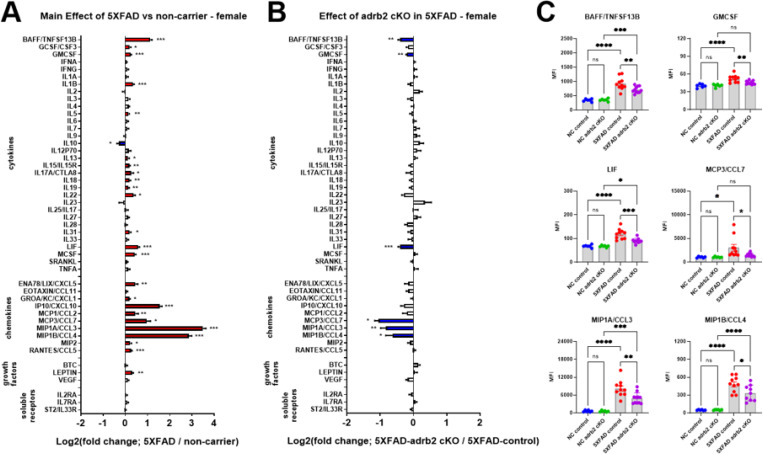
Effect of conditional knockout of adrb2 in microglia in female 5XFAD and non-carrier mice. Log2-fold change graphs depict the effects of **A)**5XFAD (5XFAD/non-carrier; n=21) and **B)**adrb2 cKO in microglia microglia(5XFAD-cKO/5XFAD-control; n=11) in female mice on a panel of inflammation-related markers. **C)** Bargraphs show raw data from proteins affected by adrb2 cKO, as indicated in panel **B**. For **A)** * indicates themain effect of 5XFAD. For **B)** and **C)** * indicates post-hoc Sidak’s comparison of means following two-way ANOVA (5XFAD x adrb2 cKO). *p < 0.05, ** p < 0.01, *** p < 0.001, **** p < 0.0001.

To determine the specific effects of microglial adrb1 and adrb2 receptor activation on inflammation in the 5XFAD model, adrb1 and adrb2 receptors were conditionally deleted in microglia of male and female 5XFAD and NC mice from 3 to 5.5 months of age (see Figure 1 for experimental design). Analysis of inflammatory markers in hippocampal containing coronal dissections from male and female 5XFAD mice revealed a common pattern of inflammatory marker upregulation in both the ADRB1 (Supplemental Figure S6A, male; Supplemental Figure S6C, female) and ADRB2 (Figure 8A, male; Figure 9A, female) cKO studies. The common set of cytokines and chemokines upregulated in 5XFAD male and female mice included: BAFF, IL1b, LIF, IP10/CXCL10, MCP3/CCL7, MIP1A/CCL3, MIP1B/CCL4, and Rantes/CCL5 (see Supplemental Tables S4, S5, S6, and S7 for ANOVA and post-hoc statistics). Conditional knockout of ADRB1 in microglia did not affect inflammation in either male or female 5XFAD mice (Supplemental Figure S6B, male; Supplemental Figure S6D, female; Supplemental Tables S6 and S7). Conditional knockout of ADRB2 in microglia had no effect in male 5XFAD mice (Figure 8B). However, in female 5XFAD mice, cKO of ADRB2 in microglia selectively attenuated a subset of markers that were upregulated in the 5XFAD control mice, including: BAFF, GMCSF, LIF, MCP3/CCL7, MIP1A/CCL3, and MIP1B/CCL4 (Figures 9B and 9C; Supplemental Tables S4 and S5 for ANOVA and post-hoc statistics).

### Effects of conditional knockout of adrb1 or adrb2 on Aβ pathology and LC neuronal counts in 5XFAD mice

**Figure 10:**
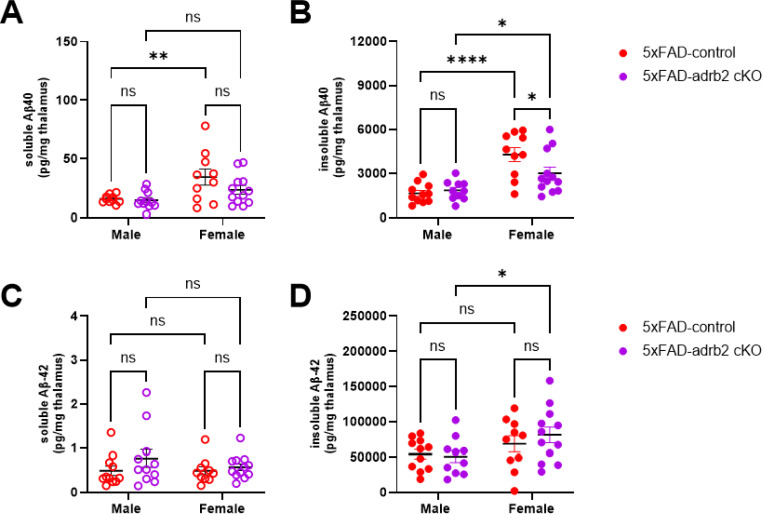
Conditional knockout of adrb2 in microglia partly reduced insoluble aβ40 in female 5XFAD mice. Bar graphs depict thalamic concentrations of soluble and insoluble **A,B)**aβ40 and **C,D)** aβ42. *p < 0.05, ** p < 0.01, ***p < 0.001, ****p < 0.0001; Sidak’s post-hoc comparison of means following two-way ANOVA (adrb2 cKO x sex).

Conditional KO (cKO) of adrb1 in microglia had no impact on soluble or insoluble Aβ40 or Aβ42 (Supplemental Figure S7). In contrast, cKO of adrb2 in microglia slightly attenuated insoluble Aβ40 in female 5XFAD mice (Figure 10B). 6E10-immunoreactivity as a marker of Aβ pathology and iba1-immunoreactivity as a marker of microglial neuroinflammation were quantified in the dentate gyrus (DG), CA3 region of the hippocampus, subiculum (SUB), and retrosplenial cortex (RS). While 5XFAD mice had elevated 6E10 in all four regions and elevated iba1 in the DG, SUB, and RS, no effects from cKO of adrb1 (Supplemental Figure S8) or adrb2 (Supplemental Figure S9) were observed for either 6E10-immunoreactivity or iba1-immunoreactivity.

TH-immunoreactive NE neurons were counted with stereology in serial sections of the LC of adrb1 cKO mice, and no effects of 5XFAD or adrb1 cKO were observed (Supplemental Figure S10; adrb2 cKO mice were not analyzed for TH-cell counts).

## Discussion

The data presented here highlight the critical role of the LC NE system in modulating neuroinflammation in a mouse model of AD. Our findings also emphasize the complexity of targeting specific adrenergic pathways in neurodegenerative diseases. Identifying the specific cell types, cellular compartments, and pathways that mediate aspects of norepinephrine function is essential to improving current treatment strategies for AD and understanding the mechanisms of action for these therapies. To explore the role of adrenergic modulation of neuroinflammation in greater detail, we used various experimental approaches to selectively modulate NE function within the 5XFAD AD mouse model. Our results support previous studies demonstrating significant neuroinflammation and amyloid-beta (Aβ) pathology in this model (11, 34, 42). We provide evidence of extensive regional Aβ accumulation and loss of tyrosine hydroxylase-immunoreactivity (TH-IR), which suggests impairment or loss of TH-expressing neurons and fibers using unbiased imaging techniques. Using chemogenetic inhibition of LC NE neurons and beta-adrenergic pharmacological inhibition using propranolol, an increase in neuroinflammation was detected in 5XFAD mice, confirming the anti-inflammatory effects of endogenous NE signaling in the brain. However, conditional deletion of beta-adrenergic receptors in microglia did not produce similar anti-inflammatory effects, indicating that receptors on other cell types, not microglia, contribute to NE’s anti-inflammatory actions in the brain. Importantly, the deletion of adrenergic receptors (adrb1 or adrb2) in microglia did not replicate the anti-inflammatory effects observed with broader pharmacological inhibition of beta-adrenergic receptors by propranolol. We did not observe effects of LC inhibition, pharmacological beta-adrenergic blockade, or microglial adrb1 or adrb2 deletion on Aβ pathology, except for a slight reduction in Aβ in females with microglial adrb2 deletion. Additionally, the profile of inflammatory markers upregulated in 5XFAD mice and their modulation by adrenergic interventions suggests the involvement of a pro-inflammatory A1 astrocyte phenotype.

Overall, our data suggest that NE’s anti-inflammatory effects in the brain may be mediated through adrenergic receptors on cell types other than microglia, potentially including astrocytes, which we plan to investigate further in future studies.

### Unbiased detection of regional Aβ pathology and microgliosis in 5XFAD

Previous studies have utilized whole-brain microscopy to map Aβ aggregation and glial reactivity in aged AD mouse models. Liebmann et al. (2016) were pioneers in using volumetric brain imaging to map pathology in AD mice aged 4 to 27 months, comparing LSFM images with conventional 2D histological methods (53). Detrez et al. (2019) mapped the spread of hyperphosphorylated tau in Tau.P301L mice and mice injected with synthetic or patient-derived tau fibrils at CA1 (54). They also visualized microglial rodlike morphological changes and reduced tau hyperphosphorylation in response to immunotherapy with anti-Tau antibody treatment (54). Canter et al. (2019) used System-Wide Control of Interaction Time and kinetics of Chemicals (SWITCH) immunolabeling for 3D mapping of Aβ deposition in 5XFAD mice (2 to 12 months of age)(55). LSFM has also been used to visualize Aβ, microglia, and astrocytes in 10-month-old 5XFAD mice (56). Gao et al. (2024) reported qualitative images of Aβ accumulation in APP knock-in, 3xTg-AD, 5XFAD, and control mice aged 6 to 17 months using iPEGASOS-based staining and clearing (57). Here, we report novel analyses to provide a detailed and rigorous quantitative description of anatomical regions with the most severe pathology in 5XFAD mice. Additionally, we comprehensively examined changes in the catecholaminergic system leveraging UNRAVEL (58), a cutting-edge LSFM analysis pipeline designed to automatically quantify features of interest while filtering out false positives and retaining sensitivity. False positives were minimized by 1) utilizing a machine learning algorithm to detect artifacts, 2) subtracting autofluorescence, 3) zeroing out voxel intensities in areas with non-specific staining (e.g., capillaries) or signal that is not of interest (e.g., resting microglia), 4) applying non-parametric permutation testing based on the general linear model for voxel-wise comparisons, 5) implementing false discovery rate correction for multiple comparisons, and 7) warping significant voxels clusters back to full-resolution atlas space to validate group differences with precise measurements of Aβ plaques, reactive microglia, TH+ fibers, and TH+ cells. Downregulation of TH in fibers was widespread, affecting the isocortex, including somatosensory, anterior cingulate, agranular insula, motor, visceral, gustatory, prelimbic, and orbital areas, cortical subplate (dorsal endopiriform nucleus, claustrum, and lateral amygdala), caudoputamen, hypothalamus, midbrain, cerebellum, and fiber tracts. A corroborating report from Baek et al. (2023), using an immuno-active clearing technique (iACT) to assess axonal projections in 5XFAD and PS19 Thy1-YFP mice, described irregular dopaminergic projections with axonal swelling, potentially linked to reduced dopamine release in the striatum (59). Our results showing decreased TH+ fibers in the temporal cortex, medial prefrontal cortex, striatum, amygdala, and hypothalamus align with abnormalities in dopaminergic and/or noradrenergic neurons. Dopaminergic inputs are prominent in the striatum, amygdala, hippocampus, prefrontal cortex, and anterior cingulate area (60), while noradrenergic projections extend across the isocortex, thalamus, hypothalamus, hippocampus, amygdala, and cerebellum. Further research is needed to differentiate the loss of dopaminergic vs. noradrenergic inputs in regions with TH+ fiber loss.

In contrast to extensive TH+ fiber loss, decreased detection of TH+ neurons was limited to specific hypothalamic nuclei and the periaqueductal grey. The reduction in TH+ projections and neurons may reflect the downregulation of TH in these compartments and/or neurodegeneration. No significant loss of LC NE neurons was detected via conventional stereology or whole-brain imaging in our brain samples studied here. Widespread TH fiber loss, compared to the minimal TH+ neuronal loss detected in this study, may suggest axonal-mediated retrograde neuronal degeneration in this model of AD. This is supported by the apparent absence of Aβ plaques and reactive microglia in areas with TH+ neuron loss.

### The pro-inflammatory effects of LC inhibition and the pharmacological blockade of beta-adrenergic receptors are not replicated by knocking out the beta-adrenergic receptors in microglia.

We demonstrate the proinflammatory effects of inhibiting LC NE neurons and blocking beta-adrenergic receptors using propranolol in 5XFAD mice, consistent with our previous report on the proinflammatory effects of beta blockers (11). Additionally, we and others have shown the anti-inflammatory effects of beta-adrenergic agonism in AD models (12, 34, 35, 61). Moreover, this is supported by previous studies in which loss of NE signaling in AD mouse models exacerbates pathology and neuroinflammation (40, 41, 62). Inhibition of the LC was achieved using inhibitory DREADD receptors in 5XFAD mice, enabling selective suppression of noradrenergic neurons in the LC to assess the impact of reduced NE tone in projection regions. We found that LC inhibition significantly increased specific inflammatory markers including BAFF, ENA78/LIX/CXCL5, MCP3/CCL7, MIP1A/CCL3, MIP1B/CCL4, and RANTES/CCL5. These markers were already upregulated in 5XFAD mice under control conditions but were further elevated following LC inhibition, suggesting an anti-inflammatory role of endogenous LC-derived NE tone on these pathways. The implications of this upregulation of an A1-inflammatory phenotype (astrocyte mediated) are discussed further below. Despite the marked increase in neuroinflammation with LC inhibition, no changes were observed in Aβ pathology, as measured by the soluble and insoluble Aβ40 and Aβ42 levels in cortical tissue.

We found that chronic administration of propranolol in 5XFAD mice exacerbated neuroinflammation, elevating specific proinflammatory cytokines and chemokines, such as IL-6, Eotaxin, and GROA/KC/CXCL1. Propranolol did not affect Aβ pathology, consistent with the DREADD LC inhibition results, suggesting that the noradrenergic system may primarily influence inflammatory pathways rather than Aβ processing. Although beta-adrenergic signaling plays a key role in regulating inflammation, its influence on Aβ deposition or clearance appears limited. Further studies are needed to assess its impact on Aβ processing and pathology in more detail.

Interestingly, conditional knockout of adrb1 or adrb2 in microglia did not produce the expected proinflammatory effects, contrasting with the results from LC inhibition and chronic administration of propranolol. Conditional deletion of adrb2 actually attenuated the inflammatory markers that were exacerbated by LC inhibition, suggesting that adrb2 on another cell type, such as astrocytes, may drive the observed neuroinflammation following the loss of NE tone. Additionally, the possibility exists that the knockout of beta-adrenergic receptors on microglia triggers compensatory upregulation on other cell types, such as astrocytes, which could explain the contrasting anti-inflammatory effects observed in our current study. This could explain why the anti-inflammatory effects of cKO of adrb2 on microglia affect the same inflammatory markers as with LC inhibition but in the opposite direction. These findings imply that the pro-inflammatory effects observed with LC inhibition and propranolol likely result from a broader disruption of NE signaling across multiple cell types. Endogenous LC-derived NE modulates neuroinflammation by acting on various adrenergic receptors throughout the brain, highlighting the complexity of NE signaling in AD-related pathophysiology.

### Adrenergic modulation of neuroinflammation is consistentwith the regulation of an A1 inflammatory response

**Figure 11:**
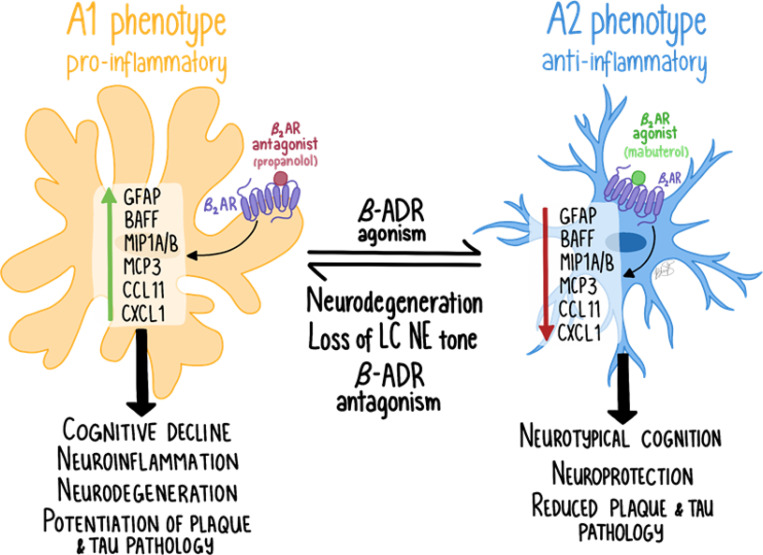
The schematic above depicts a model in which beta-adrenergic receptors (adrb, β-ADR) modulate an A1 inflammatory response in astrocytes, which is upregulated in neurodegenerative disorders and with loss of noradrenergic tone. 5XFAD mice have increased levels of A1 inflammatory markers in the brain (Figures 4A, 5A, 7A, 8A). Chemogenetic inhibition of the LC with inhibitory DREADD receptors (Figure 4B) and antagonism of β2-ADRs (Figure 5B) enhance the production of key A1-astrocytic chemokines and cytokines. Notably, conditional deletion of β2-ADR from microglia does not mimic the effect of β2-ADR antagonism or LC inhibition (Figures 7B and 8B), indicating that NE might operate through ADRs on other cell types (e.g. astrocytes) to control neuroinflammation.

A novel aspect of the current work is the identification of a selective pattern of proinflammatory cytokines and chemokines modulated across adrenergic paradigms, supporting an A1-mediated astrocytic neuroinflammatory response. The specific markers potentiated in 5XFAD mice following LC inhibition are consistent with an A1 inflammatory response in astrocytes. We detected a baseline increase in these A1 inflammatory markers in the brains of 5XFAD mice (Figures 4, 5, 8, 9), and they were further potentiated with LC inhibition (Figure 4B). These A1 markers are induced by astrocytes in various human CNS pathologies, including AD, multiple sclerosis, and CNS lymphoma (63–66). For example, MIP1B/CCL4 is linked to multiple sclerosis, post-traumatic stress disorder, and depression (67–70). MIP1A/CCL3 and eotaxin/CCL11 are linked to AD (64, 71–73), MCP1/CCL2 levels correlate with neuroinflammation, brain atrophy, and cognitive decline in AD (74–76), and MCP3/CCL7 is part of a diagnostic panel for AD (77). BAFF levels are crucial for B cell activities and are altered in Parkinson’s disease and multiple sclerosis (78–80). These findings underscore the importance of these astrocytic chemokines as biomarkers and therapeutic targets in neurodegenerative and psychiatric conditions. Notably, β2-ADRs are expressed on astrocytes in regions that receive NE enervation from the LC (31, 32, 81, 82). Thus, loss of β2-ADR tone in astrocytes following LC inhibition may unleash this A1 proinflammatory response (Figure 11). Likewise, compensatory upregulation of astrocytic b2-ADR in mice with conditional deletion of adrb2 in microglia may have resulted in the anti-inflammatory effect on the markers we observed in the cKO study.

In addition to neuroinflammation, β-ADRs on astrocytes have been shown to regulate many other critical brain functions known to be dysregulated in AD, including energy utilization (8), cerebral blood flow (CBF) (83, 84), and blood-brain barrier (BBB) integrity (18). For example, β-ADRs on astrocytes are involved in the neurovascular regulation of BBB permeability and CBF. β-ADRs on astrocytes stimulate glucose uptake, glycogen breakdown, and lactate shuttling, serving as an energy source between astrocytes and metabolically active neurons (5–7). Indeed, hypometabolism reliably precedes clinical symptoms in AD, allowing its use as a biomarker for predicting diagnosis and monitoring the progression of AD (85). β2-ADRs on astrocytes also regulate synaptic phagocytosis (86). Dysregulation of these combined systems due to loss of NE tone following LC degeneration in neurodegenerative disorders such as AD may both underlie and potentiate disease pathology. The significance of astrocytic involvement in AD is further supported by the recent identification of Disease Associated Astrocytes (DAAs) with a unique and similar genetic profile in 5XFAD mice and aged and AD human brains (87). Genes upregulated in DAAs include those encoding proteins associated with metabolic pathways, inflammatory signaling, endocytosis, the complement cascade, and Aβ metabolism and clearance (87). Finally, astrocytes play a vital role in the newly identified glymphatic system in the brain (88), thought to be essential for the clearance of Aβ and other toxins during sleep. A complete understanding of how the loss of β2-ADR tone on astrocytes may contribute to the disruption of cellular and molecular pathways that precede neurodegenerative processes is critical for a deeper understanding of the pathophysiology of AD and an area of continued research in our laboratory.

## Conclusions

This study further supports the integral role of the NE system in the regulation of neuroinflammation in the pathophysiology of AD, suggesting that the anti-inflammatory effects of NE may be mediated through adrenergic receptors on cell types other than, or in addition to, microglia, such as astrocytes. Future identification of cellular compartments and pathways mediating the effects of NE will advance the development of more precise therapeutic strategies to mitigate neuroinflammation and cognitive decline in AD.

## Supplementary Material

Supplement 1

## Data Availability

The datasets used and/or analyzed during the current study are available from the corresponding author on reasonable request.

## Highlights

1. Brain-wide, unbiased detection of amyloid beta, microgliosis, and TH downregulation in somata and fibers in the 5XFAD mouse model of AD.
2. Chemogenetic inhibition of LC potentiates neuroinflammation in 5XFAD mice.
3. Beta-blockers also potentiate neuroinflammation in this mouse model of amyloidosis
4. Neither chemogenetic inhibition via DREADD nor beta-blockers exacerbate amyloid pathology.
5. Genetic deletion of microglial adrb2 attenuates a select set of neuroinflammatory markers in female 5XFAD mice.
6. Female mice have more extensive pathology and neuroinflammation than males in the 5XFAD mouse model of AD.

## Abbreviations

6E10: monoclonal antibody for amyloid-beta
AC: Activity Chamber
AD: Alzheimer’s disease
Aβ: amyloid beta
Adrb1: beta1-adrenergic receptor
Adrb2: beta2-adrenergic receptor
APP: amyloid precursor protein
β-ADR: beta-adrenergic receptor
BAFF: B-cell activating factor
BTC: betacellulin
cc3d: connected-components-3d
CID: collision-induced dissociation
CNO: clozapine-N-oxide
CNS: central nervous system
*Cx3cr1*: C-X3-C motif chemokine receptor 1
DABCO: 1,4-Diazabicyclo[2.2.2]octane (antifade agent)
DAPI: 4’,6-diamidino-2-phenylindole dihydrochloride (nuclear marker)
DBH: dopamine beta hydroxylase locus
DIO: double-floxed inverse orientation
DREADD: Designer Receptors Exclusively Activated by Designer Drugs
DTT: dithiothreitol
eF1a: eukaryotic translation elongation factor 1 alpha
ENA-78 (CXCL5): epithelial-derived neutrophil-activating peptide 78
Eotaxin (CCL11): eosinophil chemotactic protein
FAD: familial Alzheimer’s disease
FDR: false discovery rate
FSL: FMRIB software library
G-CSF (CSF-3): granulocyte colony-stimulating factor
GM-CSF: granulocyte-macrophage colony-stimulating factor
GRO alpha (CXCL1): growth-regulated oncogene alpha
hM4Di: Gi-coupled human M4 muscarinic receptor
Iba1: ionized calcium-binding adapter molecule-1
iDISCO: immunolabeling-enabled three-dimensional imaging of solvent-cleared organs
IF: immunofluorescence
IFNA: Interferon alpha
IFNG: Interferon gamma
IL: Interleukin
IL1A: IL-1 alpha I
L1B: IL-1 beta
IL-12p70: IL-12 p70 subunit
IL-15R: IL-15 receptor
IL-17A (CTLA-8): IL-17 alpha (Cytotoxic T-Lymphocyte Antigen 8)
IL-2R: IL-2 receptor
IL-7R alpha (IL17RA): IL-7 receptor alpha (IL-17 receptor A)
IL-33R (ST2): IL-33 receptor (Suppression of Tumorigenicity 2)
IP-10 (CXCL10): Interferon gamma-induced protein 10
ir: immunoreactivity
ITI: inter-trial intervals
K3EDTA: tripotassium ethylenediaminetetraacetic acid
KEGG: Kyoto Encyclopedia of Genes and Genomes
LC: locus coeruleus
LC-MS/MS: Liquid Chromatography-Tandem Mass Spectrometry
LIF: leukemia inhibitory factor
LSFM: light sheet fluorescence microscopy
LysC: Lysyl endopeptidase
SF: macrophage colony-stimulating factor
MCP-1 (CCL2): monocyte chemoattractant protein 1
MCP-3 (CCL7): monocyte chemoattractant protein 3
MIP-1 alpha (CCL3): macrophage inflammatory protein 1 alpha
MIP-1 beta (CCL4): macrophage inflammatory protein 1 beta
MIP-2: macrophage inflammatory protein 2
NC: non-carrier
NE: norepinephrine, noradrenaline
PB: phosphate buffer
PBS: phosphate-buffered saline
PBST: phosphate-buffered saline with Tween-20
PFA: paraformaldehyde
rAAV: recombinant adeno-associated viral vectors
RANKL: receptor activator of nuclear factor kappa-Β ligand
RANTES (CCL5): Regulated upon Activation, Normal T-cell Expressed and Secreted
SDS: sodium dodecyl sulfate
TBS: tris-buffered saline
TEAB: triethylammonium bicarbonate
TH: tyrosine hydroxylase
TNFA: Tumor necrosis factor alpha
UPLC: Ultra-Performance Liquid Chromatography
UNRAVEL: UN-biased high-Resolution Analysis and Validation of Ensembles using Light sheet images
VEGF-A: vascular endothelial growth factor A
YM-FA: Y-Maze: Forced Alternation
