# Supplementary material for: Impact of noradrenergic inhibition on neuroinflammation and pathophysiology in mouse models of Alzheimer’s disease
Source: Res Sq. 2024 Oct 30:rs.3.rs-5328229. Preprint. [Version 1] doi: 10.21203/rs.3.rs-5328229/v1 (PMC11581111; doi:10.21203/rs.3.rs-5328229/v1)
Supplement: Supplement 1 [file NIHPPRS5328229V1-supplement-1.pdf]

Supplementary Files

This is a list of supplementary files associated with this preprint. Click to download.

- DREADDpropranololcKOSupplementalFiguresFINAL.docx

- DREADDpropranololcKOSupplementalTablesFINAL.docx
- VideoS13Dviewofvalidclusters.mp4
